# Oxidative stress index of porcine follicular fluid influences meiotic maturation and embryo development during *in vitro* culture

**DOI:** 10.14202/vetworld.2025.2078-2086

**Published:** 2025-07-27

**Authors:** Bin Liu, Takeshige Otoi, Zhao Namula, Oky Setyo Widodo, Maki Hirata, Aya Nakai, Qingyi Lin, Yuichiro Nakayama, Megumi Nagahara, Fuminori Tanihara

**Affiliations:** 1 Bio-Innovation Research Center, Tokushima University, 779-3233 Tokushima, Japan; 2 Division of Animal Husbandry, Faculty of Veterinary Medicine, Universitas Airlangga, 60115 Surabaya, Indonesia; 3 Department of Obstetrics, Gynaecology and Reproduction, Faculty of Veterinary Science, Chulalongkorn University, 10330 Bangkok, Thailand; 4 Department of Veterinary Medicine, College of Coastal Agricultural Sciences, Guangdong Ocean University, 524091 Zhanjiang, China

**Keywords:** embryo development, glutathione, *in vitro* maturation, oxidative stress index, porcine follicular fluid, reactive oxygen species

## Abstract

**Background and Aim:**

Porcine follicular fluid (pFF) is frequently used to mimic the follicular microenvironment during *in vitro* maturation (IVM) of oocytes. However, the influence of oxidative stress levels within pFF on oocyte quality and embryo development remains unclear. This study aimed to investigate how varying oxidative stress index (OSI) of pFF affect porcine oocyte meiotic progression, fertilization, and embryonic development during IVM.

**Materials and Methods:**

Oocytes were matured in IVM media supplemented with 30% pFF classified into low (OSI 19), medium (OSI 22), and high (OSI 25) oxidative stress groups, based on the ratio of diacron-reactive oxygen metabolites to biological antioxidant potential. Post-IVM, oocytes were assessed for meiotic stage, DNA fragmentation, reactive oxygen species (ROS), and glutathione (GSH) levels. Fertilization and embryo development outcomes were monitored following *in vitro* fertilization and culture.

**Results:**

The OSI 19 group showed significantly higher maturation to the metaphase II stage and improved fertilization and blastocyst formation rates compared to OSI 22 and OSI 25 groups (p < 0.05). ROS and GSH levels were also significantly elevated in OSI 19 oocytes, without an increase in DNA fragmentation. Blastocysts from the OSI 25 group exhibited significantly higher DNA fragmentation index than those from the OSI 19 group (p < 0.05).

**Conclusion:**

The OSI of pFF modulates porcine oocyte competence and embryonic outcomes. Lower OSI is associated with enhanced antioxidant balance, meiotic maturation, and embryo quality. Monitoring pFF oxidative status may improve assisted reproductive outcomes in swine.

## INTRODUCTION

In recent years, significant advancements have been made in the *in vitro* production of porcine embryos, enhancing their utility in agricultural biotechnology for applications such as genetic improvement, disease modeling, and reproductive research. Despite these developments, embryos produced *in vitro* still exhibit lower developmental competence compared to their *in vivo* counterparts. This discrepancy is primarily attributed to oxidative stress resulting from suboptimal culture environments and media formulations [1, 2]. Elevated levels of reactive oxygen species (ROS) can inflict oxidative damage, adversely affecting critical reproductive processes, including oocyte maturation, follicular development, steroidogenesis, and embryogenesis [1]. Thongkittidilok *et al*. [3] and Namula *et al*. [4] have shown that the inclusion of antioxidants in *in vitro* culture media can enhance embryonic development.

To assess oxidative stress, both damage markers, such as malondialdehyde, nitric oxide, and protein carbonyl, and antioxidant indicators, such as superoxide dismutase (SOD) and reduced glutathione (GSH), are commonly measured [5, 6]. More recently, the development of free radical analyzers capable of quantifying diacron-reactive oxygen metabolites (d-ROM) and biological antioxidant potential (BAP) has facilitated rapid and accurate evaluation of oxidative status. The oxidative stress index (OSI), calculated as the ratio of d-ROM to BAP multiplied by 100, has gained widespread use in clinical and preventive medicine, including in sports and reproductive health [7]. In reproductive medicine, for example, premature ovarian insufficiency has been linked to elevated serum d-ROM levels, reflecting increased oxidative damage from mitochondrial dysfunction or ROS cytotoxicity [8]. Moreover, lower OSI levels in follicular fluid have been associated with superior fertilization outcomes and enhanced embryonic development [9], underscoring the relevance of oxidative balance in oocyte competence.

Porcine follicular fluid (pFF) is frequently incorporated into *in vitro* maturation (IVM) media to better replicate the *in vivo* follicular environment. Its inclusion has been shown to stimulate cumulus cell expansion, increase intracellular GSH concentrations in oocytes, and improve subsequent fertilization and embryo development rates [10]. The antioxidant properties of pFF, largely due to its SOD isoenzymes, are believed to shield oocytes from ROS-induced damage and support their maturation, thereby enhancing embryonic potential [11].

While pFF is widely used to support oocyte maturatio*n in vitro*, the inherent variability in its biochemical composition poses a challenge for standardization in embryo production systems. A critical yet underexplored component of this variability is the OSI, which reflects the balance between ROS and antioxidant defenses in the follicular microenvironment. Although previous studies have demonstrated the beneficial effects of antioxidant supplementation on IVM and embryo development, few have investigated the physiological oxidative status of the pFF itself and how its intrinsic OSI levels influence oocyte quality, fertilization success, and blastocyst development. Furthermore, while elevated OSI levels have been linked to reduced oocyte competence in human-assisted reproductive technology, this relationship remains poorly characterized in livestock species such as pigs. Existing data are also limited by methodological inconsistencies, including the use of low pFF concentrations that may dilute the measurable effects of oxidative stress. Consequently, the influence of pFF OSI on porcine reproductive outcomes remains largely unquantified, and a mechanistic understanding of this relationship is lacking.

The present study aimed to systematically evaluate the impact of pFF OSI on the meiotic progression, fertilization capacity, and embryonic developmental competence of porcine oocytes during IVM. By categorizing pFF into low, medium, and high OSI groups based on d-ROM and BAP measurements, we assessed their respective effects on oocyte maturation, intracellular ROS and GSH levels, DNA fragmentation, fertilization outcomes, and blastocyst quality. This investigation sought to determine whether OSI can serve as a predictive biomarker for oocyte competence and to provide empirical evidence for optimizing pFF selection in IVM protocols. Ultimately, our findings aim to improve the consistency and success of *in vitr*o embryo production in swine, with potential applications in both agricultural biotechnology and reproductive medicine.

## MATERIALS AND METHODS

### Ethical approval

Ethical approval was not required because no live animals were used in this study.

### Study period and location

The study was conducted from February 2024 to May 2024 at the Bio-Innovation Research Center, Tokushima University.

### Sample collection and preparation of pFF

Porcine ovaries of prepubertal crossbred gilts (Landrace × Large White × Duroc breeds) were obtained from a local slaughterhouse in Tokushima, immersed in 0.9% sterile saline at 30°C–33°C, and transported to the laboratory within 2 h. Follicular fluid was aspirated from 3 mm to 15 mm diameter follicles, which were typically not classified as cystic, using a 5 mL syringe fitted with an 18-gauge needle and pooled into a sterile container. The collected pFF was centrifuged at 1,710 × *g* for 60 min at 4°C, and the supernatant was harvested and stored at −30°C until use (approximately 1 month). Although hormone levels (e.g., estrogen and progesterone) in the pFF were not directly measured, samples exhibiting blood contamination, turbidity, or clot formation were excluded based on visual evaluation.

### OSI grouping

**Figure 1:**
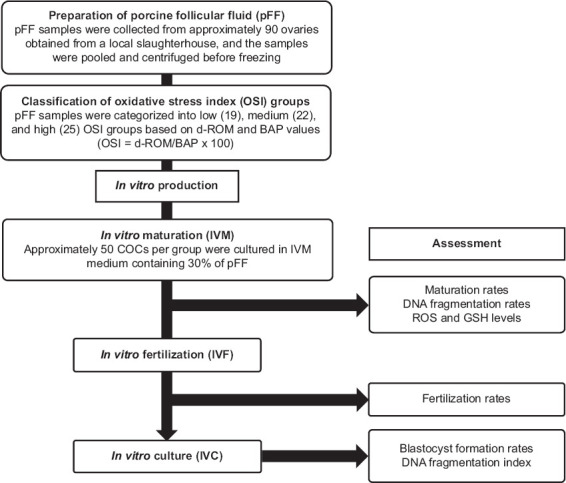
Experimental flowchart of porcine follicular fluid supplementation and oocyte evaluation in *in vitro* maturation and fertilization.

Variability in oxidant and antioxidant levels among pFF samples may arise from factors such as animal health status, environmental conditions, and transportation [12]. To account for this variability, the pFF samples were categorized into three groups based on OSI: Low, medium, and high. A total of 13 pooled pFF samples were randomly prepared from approximately 90 porcine ovaries collected over multiple days. d-ROM and BAP values were measured in each pooled sample, yielding OSI values ranging from 18.7 to 24.7. To establish the low, medium, and high OSI groups, pooled samples with similar OSI values were combined and reassessed, resulting in three defined groups with OSI values of 19.0 (low), 21.6 (medium), and 25.2 (high). Each OSI group comprised four to five pooled samples, corresponding to approximately 25–35 ovaries per group. Although a formal power analysis was not performed to determine the number of pFF pools, group sizes were chosen to balance biological variability with logistical feasibility while maintaining distinct OSI ranges between groups. When comparing OSI levels in IVM media supplemented with either 10% or 30% (v/v) pFF, significant differences were observed at 30% (e.g., 12.8 ± 0.2 for OSI 19 and 14.5 ± 0.5 for OSI 25), whereas differences were not significant at 10%, likely due to dilution effects (7.1 ± 0.3 for OSI 19 and 8.1 ± 0.2 for OSI 25). Although 10% pFF is commonly used in IVM, a 30% concentration was selected in this study to better distinguish the effects of varying OSI levels (Figure 1).

### Assessment of oxidant and antioxidant stress levels in pFF

The oxidative and antioxidative capacities of each pFF sample were assessed by measuring d-ROM and BAP using a free radical analyzer (Redoxlibra, Wismerll Co., Ltd., Tokyo, Japan), following the protocol by Hashimoto *et al*. [13]. The OSI was subsequently calculated as the d-ROM-to-BAP ratio multiplied by 100. Briefly, to measure d-ROM levels using ad-ROM test kit (Wismerll Co., Ltd., Japan), 20 μL of the pFF was added to a cuvette containing 1.2 mL of acidic buffer (pH 4.8) with iron ions, followed by 20 μL of a coloring solution containing N, N-diethyl-p-phenylenediamine. The mixture was gently inverted for homogenization. The hydroperoxide concentration was then determined by measuring the absorbance at 505 nm using a free radical analyzer. Results were expressed in Carratelli units (U.CARR), where 1 U.CARR corresponds to 0.08 mg of H_2_O_2_ per dL. To assess BAP levels using the BAP test kit (Wismerll Co., Ltd.), 50 μL of a trivalent iron-containing coloring solution was added to a cuvette containing 1.0 mL of thiocyanate, and the mixture was mixed by inversion. The initial absorbance was then recorded at 505 nm. Subsequently, 10 μL of the pFF sample was added to the cuvette and mixed again by inversion. A second absorbance reading was taken, and the change in absorbance was calculated to determine the BAP value, expressed as μmol/L of reduced ferric ions. To ensure technical accuracy, all d-ROM and BAP measurements were conducted in duplicate following calibration with a standard reference solution, and the mean values were calculated.

### Oocyte maturation

IVM was conducted with minor modifications to the protocol described by Namula *et al*. [4], notably altering the pFF concentration. Cumulus-oocyte complexes (COCs) were harvested from the ovaries of prepubertal crossbred gilts (Landrace × Large White × Duroc), obtained from a local slaughterhouse. Surface follicles were dissected in sterile conditions using a surgical blade. COCs were selected based on the presence of uniformly dark cytoplasm and compact, multilayered cumulus cells under microscopic examination. Approximately 50 COCs per group were cultured for 22 h in one well of four-well dishes containing maturation medium composed of Tissue Culture Medium-199 with Earle’s salts (Thermo Fisher Scientific, MA, USA), 30% pFF corresponding to each OSI group, 50 mM sodium pyruvate (Sigma-Aldrich, St. Louis, MO, USA), 2 mg/mL D-sorbitol (Fujifilm Wako Pure Chemical Corp., Osaka, Japan), 10 IU/mL equine chorionic gonadotropin, 10 IU/mL human chorionic gonadotropin (Kyoritsu Seiyaku, Tokyo, Japan), and 50 mg/mL gentamicin (Sigma-Aldrich). After the initial culture, the COCs were transferred to a maturation medium without gonadotropic hormones and incubated for an additional 22 h at 39°C in a humidified incubator with 5% CO_2_. All maturation media were prepared using the same batch of TCM-199 and supplements to ensure batch consistency across groups. The experimenter conducting the culture and evaluation procedures was blinded to the OSI group assignments to minimize bias.

### Analysis of meiotic stage and DNA damage in oocytes

After IVM, approximately 20 oocytes per group were evaluated for nuclear status and DNA fragmentation in eight independent replicates using a combined nuclear staining and terminal deoxynucleotidyl transferase (TdT)-mediated deoxyuridine triphosphate (dUTP) nick-end labeling (TUNEL) method, as described by Thongkittidilok *et al*. [3]. In brief, oocytes were denuded from cumulus cells using 150 IU/mL hyaluronidase (Sigma-Aldrich) and subsequently fixed in 4% paraformaldehyde in phosphate buffer (Fujifilm Wako Pure Chemical Corp.) at 4°C overnight. Following fixation, the oocytes were permeabilized with 0.1% Triton X-100 (Sigma-Aldrich) for 40 min at room temperature. The oocytes were incubated overnight at 4°C in phosphate-buffered saline (PBS) supplemented with 10 mg/mL bovine serum albumin (Sigma-Aldrich). The oocytes were then incubated with fluorescein-conjugated 2′-deoxyuridine-5′-triphosphate (fluorescein-dUTP) and TdT (Roche Diagnostics Corp., Tokyo, Japan) for 1 h at 38.5°C. For positive controls, oocytes were treated with 50 μg/mL deoxyribonuclease I (DNase I) (DNase I; Sigma-Aldrich) for 20 min at 38.5°C before TUNEL staining. Negative controls were incubated with fluorescein-dUTP without the addition of TdT enzyme. Following TUNEL staining, oocytes were counterstained for 10 min with 1 mg/mL 4′,6-diamidino-2-phenylindole (DAPI) (DAPI; Thermo Fisher Scientific) for 10 min. Oocytes were mounted on glass slides using antifade mounting medium (Slow-Fade; Molecular Probes Inc., Eugene, OR, USA) and sealed with clear nail polish. Stained oocytes were visualized using an epifluorescence microscope (Eclipse 80i; Nikon, Tokyo, Japan) with a 20× objective lens. The exposure times were <10 s for both DAPI and TUNEL. Oocytes were classified into meiotic stages – germinal vesicle, condensed chromatin, metaphase I, anaphase I–I-telophase I (AT), and metaphase II (MII) – based on DAPI-stained chromatin morphology. DNA fragmentation was assessed by quantifying TUNEL-positive nuclei in each oocyte.

### Evaluation of ROS and GSH levels in porcine oocytes

ROS and GSH levels in oocytes following IVM were measured according to the protocol of Jeon *et al*. [14]. Briefly, after 44 h of IVM, oocytes were collected and denuded of cumulus cells using hyaluronidase. Both ROS and GSH measurements were performed using approximately 30 oocytes per group, with the same number used in each replicate to ensure consistency. Approximately 30 oocytes per group were incubated in IVM medium containing 10 μM 2′,7′-dichlorodihydrofluorescein diacetate (H2DCFDA) (H2DCFDA; D399, Thermo Fisher Scientific) for ROS detection or 10 μM 4-chloromethyl-6,8-difluoro-7-hydroxycoumarin (CMF2HC) (CMF2HC; C12881, Thermo Fisher Scientific) for GSH detection, for 15or 30min, respectively. Following incubation, the oocytes were washed with 0.1% (w/v) polyvinyl alcohol-PBS and transferred to 10-μL microdroplets for imaging. Fluorescence signals were visualized using an Eclipse 80i, where green fluorescence indicated ROS and blue fluorescence indicated GSH levels. The captured images were stored as Tagged Image File Format (TIFF) files for subsequent analysis. The fluorescence intensity of each oocyte was quantified using ImageJ software (version 1.54 g; National Institutes of Health, Bethesda, MD, USA). The fluorescence intensity was measured by selecting a region of interest (ROI) encompassing the entire oocyte area. Background correction was performed by subtracting the mean fluorescence intensity from ROIs in oocyte-free regions across all samples.

### *In vitro* fertilization (IVF) and assessment

Following IVM, IVF was conducted according to the protocol established by Namula *et al*. [4]. In brief, frozen-thawed spermatozoa were suspended in 5 mL of porcine fertilization medium (PFM) (PFM; Research Institute for the Functional Peptides Co., Yamagata, Japan) and centrifuged at 500 × *g* for 5 min to remove cryoprotectants. Approximately 50 mature oocytes were placed in a four-well culture dish containing 500 μL of sperm-supplemented PFM. Oocytes were co-incubated with the frozen-thawed spermatozoa (1 × 10^6^ sperm/mL) in PFM for 5 h at 39°C in a humidified incubator maintained at 5% CO_2_, 5% O_2_, and 90% N_2_. The frozen-thawed spermatozoa for IVF were collected from a Duroc boar at the Tokushima Prefectural Livestock Research Institute (Tokushima, Japan). Semen samples were selected based on achieving a total fertilization rate of over 60% in preliminary IVF trials. To assess fertilization, a subset of oocytes was fixed 10-h post-insemination in a 1:3 (v/v) acetic acid: ethanol solution for 48–72 h. Fixed oocytes were stained with 1% acetic orcein in 45% acetic acid and examined using phase-contrast microscopy. Fertilized oocytes were identified by the presence of both male and female pronuclei and were further classified as normal or polyspermic based on the number of pronuclei or cytoplasmic swollen sperm heads. The monospermic fertilization rate was calculated as the number of monospermic oocytes divided by the total number of fertilized oocytes.

### Embryo culture

After IVF, the presumptive zygotes were washed and cultured in groups within four-well dishes containing porcine zygote medium (PZM) (PZM-5; Research Institute for the Functional Peptides Co.), overlaid with mineral oil, and incubated at 39°C in a humidified atmosphere of 5% CO_2_, 5% O_2_, and 90% N_2_ for 72 h [4]. At 72-h post-insemination, only cleaved embryos were selected and transferred to porcine blastocyst medium (PBM; Research Institute for Functional Peptides Co.). The selected embryos were cultured for an additional 4 days to evaluate their progression to the blastocyst and expanded blastocyst stages.

### Blastocyst assessment

Upon reaching the blastocyst stage, DNA fragmentation in embryos with ≥20 cells was assessed using the TUNEL assay, as previously described. The DNA fragmentation index was determined by dividing the number of TUNEL-positive nuclei by the total number of nuclei per blastocyst. The number of cleaved embryos, number of blastocysts, total blastocyst cell count, and DNA fragmentation index were recorded.

### Statistical analysis

Each experimental group was replicated in eight biological trials using oocyte samples that were independently collected. Statistical analysis was conducted using one-way analysis of variance (ANOVA), followed by Fisher’s protected least significant difference *post hoc* test, with STATVIEW (Abacus Concepts, Inc., Berkeley, CA, USA). Percentage data of oocyte maturation, fertilization, and embryonic development were subjected to arcsine square root transformation before statistical testing. The normality of data distribution was evaluated using the Jarque–Bera test before performing ANOVA. Results are presented as mean ± standard error of the mean. The values presented in the figures and tables were back-transformed to percentage values after statistical analysis. Statistical significance was defined as p ≤ 0.05.

## RESULTS

### Meiotic maturation outcomes

As presented in Table 1, the proportion of oocytes reaching the MII stage following IVM was significantly greater in the OSI 19 group (p = 0.0008) and OSI 22 group (p = 0.0089) compared to the OSI 25 group, with a 95% confidence interval (CI) of 60.51–70.01. The percentage of DNA-fragmented nuclei in oocytes post-IVM did not differ significantly among the OSI groups.

**Table 1:** Effects of porcine follicular fluid (pFF) with different oxidative stress index (OSI) values on the meiotic competence and DNA integrity of porcine oocytes.

Group*	Number of examined oocytes	Number (%) of oocytes with		Number (%) of oocytes with DNA-fragmented nuclei

		GVBD	MII	
OSI 19	162	152 (93.9 ± 1.2)^b^	118 (72.8 ± 3.6)^a^	2 (1.3 ± 0.8)
OSI 22	164	161 (98.1 ± 0.9)^a^	112 (68.4 ± 3.3)^a^	3 (1.8 ± 0.9)
OSI 25	163	146 (89.7 ± 2.6)^b^	89 (54.6 ± 2.9)^b^	2 (1.3 ± 0.8)

* The OSI levels of mixed pFF were 19.0, 21.6, and 25.2 for OSI 19, OSI 22, and OSI 25, respectively. GVBD=Germinal vesicle breakdown, MII=Metaphase II. ^a,b^Values with different superscript letters in the same column are significantly different (p < 0.05)

### Intracellular oxidative markers

**Figure 2:**
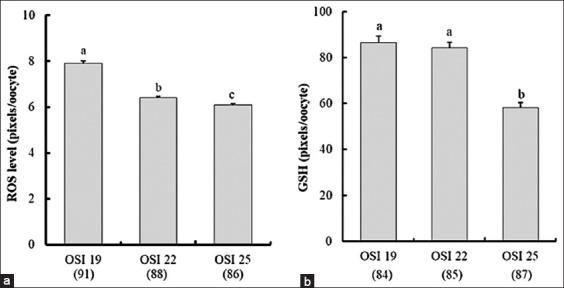
Effects of porcine follicular fluid (pFF) supplementation with different oxidative stress index (OSI) values on (a) reactive oxygen species and (b) glutathione levels in *in vitro* matured porcine oocytes. The OSI values of mixed pFF were 19.0, 21.6, and 25.2 for groups OSI 19, OSI 22, and OSI 25, respectively. The bars with different letters (a–c) represent significant differences (p < 0.05) as determined by one-way analysis of variance followed by Fisher’s protected least significant difference *post hoc* test. The numbers in parentheses indicate the number of examined oocytes.

As illustrated in Figure 2, oocyte ROS levels declined significantly as OSI values increased (95% CI, 6.69–6.95, p < 0.0001), with the highest ROS levels observed in the OSI 19 group. In addition, GSH concentrations in oocytes were significantly elevated in the OSI 19 and OSI 22 groups compared to the OSI 25 group (95% CI, 72.91–79.36, p < 0.0001).

### Fertilization and blastocyst formation

Table 2 shows that fertilization rates were significantly higher in the OSI 19 group compared to the OSI 22 group (p = 0.0248) and OSI 25 group (p = 0.0043), with a 95% CI of 51.18–58.72. Similarly, the OSI 19 group exhibited a significant increase in blastocyst formation rates compared to the OSI 22 group (p = 0.0312) and OSI 25 group (p = 0.0032), with a 95% CI of 5.51–8.57.

**Table 2:** Effects of porcine follicular fluid (pFF) with different oxidative stress index (OSI) values on the fertilization, development, and DNA integrity of porcine embryos.

Group*	Number of examined oocytes	Number (%) of oocytes**		Number of examined oocytes	Number (%) of embryos		Total number of blastocyst cells	DNA- fragmentation index (%)
	
		Fertilized	Monospermy		Cleaved	Developed for blastocysts		
OSI 19	121	75 (62.3 ± 2.9)^a^	54 (72.0 ± 3.6)	277	215 (77.8 ± 2.4)^a^	28 (10.1 ± 1.3)^a^	37.5 ± 1.6	17.6 ± 0.6^a^
OSI 22	117	62 (53.0 ± 2.1)^b^	50 (80.6 ± 2.3)	313	218 (69.9 ± 4.8)^a,b^	20 (6.4 ± 0.9)^b^	40.7 ± 1.9	18.2 ± 0.9^a^
OSI 25	113	56 (49.6 ± 3.4)^b^	41 (72.9 ± 6.1)	323	211 (65.3 ± 2.9)^b^	15 (4.7 ± 1.2)^b^	42.9 ± 2.7	21.3 ± 1.0^b^

* The OSI levels of mixed pFF were 19.0, 21.6, and 25.2 for OSI 19, OSI 22, and OSI 25, respectively.

** Fertilization rate was defined as the ratio of the number of fertilized oocytes to the total number of examined oocytes. The monospermic fertilization rate was defined as the ratio of the number of monospermic oocytes to the total number of fertilized oocytes. ^a,b^Values with different superscript letters in the same column are significantly different (p < 0.05)

### Blastocyst quality assessment

The total number of blastocyst cells did not differ significantly among the three OSI groups. However, the DNA fragmentation index of blastocysts in the OSI 25 group was significantly higher (95% CI, 17.70–19.68) than in the OSI 19 group (p = 0.028) and OSI 22 group (p = 0.162).

## DISCUSSION

### Overview of pFF oxidative status and oocyte development

This study evaluated the influence of pFF with varying OSI on oocyte maturation and embryonic development during IVM. Our findings demonstrated that the oxidative status of pFF significantly influenced both meiotic progression and embryonic development of porcine oocytes (Table 3). Follicular fluid is composed of a complex array of biomolecules, including steroids, metabolites, polysaccharides, proteins, low-molecular-weight peptides, ROS, and antioxidant enzymes [15]. Beyond their signaling functions, these molecular interactions are believed to facilitate optimal follicular growth and oocyte maturation [16]. A comparative study reported that pFF exhibited 7.2-fold greater SOD activity and enhanced radical-scavenging capacity relative to fetal bovine serum during porcine oocyte maturation [11]. Thus, the antioxidant properties of pFF may mitigate oxidative damage by scavenging ROS, thereby enhancing oocyte meiotic competence.

**Table 3:** Summary of developmental parameters across different oxidative stress index (OSI) levels.

Indicator	OSI 19	OSI 22	Trend*	OSI 25	Trend*
MII (%)	72.8	68.4	-	54.6	↓
ROS level	7.9	6.4	↓	6.1	↓
GSH level	86.6	84.2	-	58.2	↓
Total IVF (%)	62.3	53.0	↓	49.6	↓
Blastocyst formation (%)	10.1	6.4	↓	4.7	↓
DNA fragmentation index (%)	17.6	18.2	-	21.3	↑

* Compared with OSI19, arrows (↓ or ↑) indicate a decreasing or increasing trend, respectively. The dashed line (–) indicates no obvious or statistically significant changes. ROS=Reactive oxygen species, GSH=Glutathione, IVF=*In vitro* fertilization, MII=Metaphase II

### Effect of OSI on meiotic maturation, fertilization, and embryo development

In this study, oocyte maturation rates at the MII stage were significantly lower in the high-OSI (OSI 25) group than in the low-OSI (OSI 19) group. In addition, the OSI 19 group exhibited significantly higher fertilization and blastocyst formation rates than the medium- and high-OSI groups. These results are consistent with human studies, which show that oocytes from low-OSI follicles are associated with improved fertilization outcomes and embryo development.

### Role of ROS and GSH in oocyte competence

Notably, ROS levels in oocytes were highest in the low-OSI group and progressively decreased with increasing OSI values, mirroring the trends observed in maturation, fertilization, and blastocyst formation rates. The underlying mechanism responsible for the observed elevated ROS levels in oocytes from the low-OSI group remains uncertain. While excessive ROS levels are often associated with cellular damage, moderate levels play essential roles in signal transduction, gene regulation, and early embryonic development [15]. Specific oxidative pathways may modulate oocyte competence, and a defined oxidative balance in follicular fluid may be necessary to support optimal maturation [17]. Overall, a moderate balance of oxidative stress is necessary to maintain homeostasis *in vivo* through the antioxidant response to excess ROS [18].

Conversely, in contrast to the high-OSI group, the low-OSI group exhibited elevated GSH levels without a corresponding increase in DNA fragmentation. GSH is vital for neutralizing intracellular ROS, protecting lipids and DNA from oxidative damage, and enhancing oocyte antioxidant capacity by regulating related enzyme systems [19]. Adequate GSH concentrations are essential for oocyte maturation, as elevated ROS levels correlate with diminished oocyte quality and apoptosis [20]; moreover, GSH is consumed through interaction with ROS. In addition, excessive ROS may stimulate the expression of antioxidant-responsive genes, thereby upregulating GSH synthesis. However, insufficient GSH levels may intensify oxidative stress, promote ROS accumulation, and induce mitochondrial dysfunction [21]. The interplay between GSH and ROS highlights GSH’s crucial role in mitigating oxidative damage, thereby preserving oocyte integrity and promoting reproductive success. This mechanistic relationship may explain the superior developmental competence observed in the low-OSI group despite elevated ROS concentrations.

### DNA integrity of the blastocysts

Blastocysts derived from the low-OSI group exhibited a significantly lower DNA fragmentation index than those from the high-OSI group. As a major non-enzymatic antioxidant, GSH is a key indicator of cytoplasmic maturation and oocyte quality at the completion of IVM [22]. Elevated GSH levels in bovine oocytes have also been shown to enhance fertilization rates and support advanced embryonic development *in vitro* [23].

### Possible mechanisms underlying OSI effects

The OSI of pFF used during IVM was shown to significantly influence the meiotic and developmental competence of porcine oocytes. Our results suggest that increased GSH levels in the low-OSI group enhance oocyte antioxidant capacity, thereby improving developmental competence and embryonic quality following IVF.

### Limitations and future directions

This study had several limitations. First, despite performing eight biological replicates, the modest sample size might limit the statistical power of the study. Second, the *in vitro* culture conditions, including endocrine and paracrine factors, did not fully replicate the complex *in vivo* follicular environment. Third, while the study assessed phenotypic endpoints such as maturation rates, ROS/GSH levels, and embryonic development, it did not evaluate molecular markers related to oxidative stress responses or gene expression. Assays like quantitative PCR would provide deeper mechanistic insights. Fourth, although trends suggest a relationship between OSI levels and developmental competence, the underlying mechanisms remain unclear. While elevated GSH levels in the low-OSI group may enhance oocyte resilience, direct evidence for this is currently lacking. Prior studies by Shadel and Horvath [24] and Sinenko *et al*. [25] have indicated that moderate ROS levels can stimulate development, whereas excessive ROS may impair mitochondrial function. Given these considerations, and despite the significant associations between pFF OSI levels and oocyte/embryo outcomes, we have limited our interpretation to association rather than causation. Therefore, future studies integrating molecular profiling are required to establish causal relationships and elucidate these mechanisms.

## CONCLUSION

This study demonstrated that the OSI of pFF plays a critical role in regulating the meiotic maturation and developmental competence of porcine oocytes during IVM. Oocytes exposed to low-OSI (OSI 19) pFF showed significantly higher maturation to the MII stage, elevated intracellular ROS and GSH levels, improved fertilization and blastocyst formation rates, and reduced DNA fragmentation in resulting blastocysts, compared to those treated with medium (OSI 22) or high-OSI (OSI 25) pFF.

The findings emphasize the importance of assessing and optimizing the redox status of pFF before its use in IVM protocols. Using pFF with a favorable OSI could enhance oocyte quality and embryonic development in porcine IVF systems, which is critical for improving the efficiency of assisted reproductive technologies in animal production and biomedical research.

A major strength of this work lies in its systematic evaluation of pFF OSI effects using well-defined biochemical markers (d-ROM, BAP), paired with functional outcomes including meiotic progression, oxidative status, fertilization capacity, and blastocyst quality. The study used a robust design with eight biological replicates, blinded evaluations, and consistent media preparation to ensure reliability.

In summary, the oxidative balance of pFF has a significant influence on porcine oocyte maturation and embryo development *in vitro*. Monitoring and modulating OSI may represent a practical, non-invasive strategy to improve IVM outcomes. These findings offer a foundation for optimizing embryo production systems and advancing reproductive biotechnologies in swine and potentially other mammalian species.

## DATA AVAILABILITY

All the generated data are included in the manuscript.

## AUTHORS’ CONTRIBUTIONS

BL and ZN: Performed the experiments and drafted the manuscript. MN: Designed the study and revised the manuscript. QL, MH, and YN: Performed the laboratory work and statistical analyses. OSW, AN, and FT: Supervised the study and reviewed the manuscript. TO: Coordinated all experiments and reviewed the manuscript. All authors have read and approved the final version of the manuscript.
